# Inflammation in Viral Vector-Mediated Ocular Gene Therapy: A Review and Report From a Workshop Hosted by the Foundation Fighting Blindness, 9/2020

**DOI:** 10.1167/tvst.10.4.3

**Published:** 2021-04-02

**Authors:** Ying Kai Chan, Andrew D. Dick, Sara Mary Hall, Thomas Langmann, Curtis L. Scribner, Brian C. Mansfield, for the Ocular Gene Therapy Inflammation Working Group

**Affiliations:** 1 Harvard Medical School, Boston, MA, USA; 2 UCL Institute of Ophthalmology, London, UK; 3 University of Bristol, Bristol, UK; 4 Hubble Therapeutics, Boston, MA, USA; 5 University Hospital, Cologne, Germany; 6 Oakland, CA, USA; 7 Foundation Fighting Blindness, Columbia, MD, USA

**Keywords:** retina, gene therapy, inflammation, immunosuppression, efficacy

## Abstract

On September 14–15, 2020, the Foundation Fighting Blindness convened a virtual workshop to discuss intraocular inflammation during viral vector-mediated gene therapy for inherited retinal diseases. The workshop's goals were to understand immune activation's nature and significance during ocular gene therapy, consider whether ocular inflammation limits gene therapy's potential, and identify knowledge gaps for future research. The event brought together a small group of experienced researchers in the field to present and discuss current data. Collectively, participants agreed that clinical, as well as subclinical, inflammation during ocular gene therapy is common. The severity of inflammation in both animal and clinical studies varied widely but is generally related to vector dose. Severe inflammation was associated with reduced gene therapy efficacy. However, the relationship between outcomes and subclinical inflammation, pre-existing antivector antibodies, or induced adaptive immune responses is still unclear. Uncertainties about the contribution of vector manufacturing issues to inflammation were also noted. Importantly, various immunosuppressive treatment protocols are being used, and this heterogeneity confounds conclusions about optimal strategies. Proposed near-term next steps include establishing an immunological consultant directory, establishing a data repository for pertinent animal and clinical data, and developing a larger meeting. Priority areas for future research include deeper understanding of immune activation during retinal diseases and during ocular gene therapy; better, harmonized application of animal models; and identifying best practices for managing gene therapy vector-related ocular inflammation.

**Translational Relevance:**

Subclinical or clinical inflammation often arises during ocular gene therapy with viral vectors. Understanding the biological bases and impacts on efficacy are important for clinical management and the improvement of future therapies.

## Overview of the Workshop

On September 14–15, 2020, the Foundation Fighting Blindness convened a workshop to discuss intraocular inflammation that may be caused by viral vector-mediated gene therapy for inherited retinal diseases. The starting point for this event arose from informal conversations among a small group of attendees during the 2019 Retinal Cell and Gene Therapy Innovation Summit, which was held immediately before the Association for Research in Vision and Ophthalmology (ARVO) annual meeting. The group noted that inflammation seemed common during treatments, but few meeting presentations addressed its implications: vector doses appeared to be chosen primarily to avoid strong inflammatory responses, but potentially at the expense of using optimal therapeutic amounts. To address this significant issue, the Foundation assembled an organizing committee to develop a structured workshop that would focus attention on this important topic. To establish the current landscape of awareness and treatment of inflammation in viral gene therapy of the retina, participants responded to a premeeting survey (see survey questions and results summary online, under “Analysis of the Pre-Meeting Survey”). Although initially planned as an in-person meeting, the meeting was conducted virtually due to COVID-19 pandemic restrictions. To conserve meeting time and establish a common background, three presentations that had been intended as plenary sessions for the in-person meeting were recorded for review before the event (see below); these are now available online. The virtual meeting consisted of data presentations from preclinical and clinical research, coupled with questions for discussion. This report summarizes the workshop's deliberations and outcomes.

For this event, “inflammation” was broadly defined.^1^ The traditional concept of inflammation as outlined thousands of years ago—redness, warmth, pain, and swelling—continues today in clinical evaluations of acute inflammation. From the molecular and cellular perspective, inflammation results after invasion by pathogens or damage to tissues. Inflammatory responses can be initiated by receptors expressed by myeloid cells (including monocytes, macrophages, neutrophils, and dendritic cells), as well as by lymphocytes, epithelial cells, and fibroblasts. These pattern recognition receptors engage molecules with pathogen-associated molecular patterns and damage-associated molecular patterns, activating a variety of signal transduction pathways to trigger complex immunological and physiological responses. From the perspective of “invading” gene therapy viral vectors therefore, inflammatory host reactions range from nonspecific myeloid cell–mediated reactions via cytokine and chemokine release, to local and systemic antigen-specific antibody and T-cell responses. In the eye, inflammatory reactions include subclinical intracellular responses that lead to pathological changes in metabolism and cell survival, as well as more profound immune activation that results in clinically apparent inflammation. The workshop considered the potential for any and all of these responses to limit successful ocular gene therapy.

## Meeting Goals in the Context of Ocular Gene Therapy History and Background

Gene therapies predominantly use viral vectors for in vivo delivery of genes to augment or repair dysfunctional inherited genes. To date, more than 300 genes and loci have been implicated in causing inherited retinal diseases (IRDs), which are mostly monogenic, with varying inheritance patterns. Many of these single-gene defects may be amenable to gene therapy strategies, and efforts to develop gene-based treatments have been underway for more than 30 years. The approval of Kymriah, which uses ex vivo delivery of a chimeric antigen receptor via a lentiviral vector (Oxford BioMedica, Oxford, UK/Novartis, Basel, Switzerland) to patient T cells, validated the power of the approach. But clinical success of in vivo administration of a gene therapy has only come recently. The approval of Luxturna (voretigene neparvovec-rzyl) in 2017 to treat IRDs caused by biallelic pathogenic variants of the *RPE65* gene was a major milestone. Subretinal administration of Luxturna delivers wild-type cDNA encoding RPE65 directly to the subretinal region of diseased eyes, thereby improving functional vision.

But development of gene therapies has also been accompanied by substantial risks and tragic setbacks. In 1999, Jesse Gelsinger died after administration of an adenoviral vector to treat an X-linked metabolic disease, ornithine transcarboxylase deficiency.^2^ Mr. Gelsinger's death from multiple organ failure was attributed to severe antivector immune responses. In the early 2000s, several children being treated for X-linked severe combined immunodeficiency with a retroviral vector, which was derived from the Moloney Murine Leukemia Virus, had development of T-cell leukemias.^3^ This outcome was likely due to oncogene activation at the site of retroviral integration. These past lessons served as cautionary examples and prompted significant changes to the viral vector platforms themselves.

The field's focus also shifted to ocular gene therapy, in part because of the relative “immune privilege” of the eye, the prevalence of monogenic diseases that could be effectively addressed, decreased vector quantities needed and manufacturing costs, and the limited systemic exposure and immune responses. The ultimate goal of treating IRDs in the eye by gene therapy is now to restore functional vision, although any therapy that slows or stops disease progression and improves vision is valuable. Approaches to correcting genetic defects now include gene augmentation, which introduces a wild-type copy of the affected gene to augment expression; gene correction, through various editing techniques to correct pathogenic variants or engineer out inappropriate splicing; and gene expression modification, using techniques such as genetic knockdown of dominant negative variants. The workshop focused on gene augmentation approaches.

Although gene therapies based on lentiviral and nonviral vectors delivery are in development, vectors derived from adeno-associated virus (AAV) are currently the most commonly used platform for delivery of ocular gene therapy.^4^^,^^5^ Wild-type AAV is a small DNA virus with many naturally occurring capsid serotypes, which can be used directly for gene therapy or first intentionally modified. The virus is primarily non-integrating but persists as episomal DNA. The recombinant AAV vectors used for gene delivery have been engineered such that the only viral contents of the virion delivered to the patient are the two inverted terminal repeats (∼145 bases) required for viral formation that flank the gene of interest. The transgene is placed under the control of selected regulatory elements that include enhancers, promoters, introns, poly(A) signals, and posttranscriptional elements (e.g*.*, the Woodchuck Hepatitis Virus Posttranscriptional Regulatory Element). The important properties of the resulting recombinant AAV vector are its ability to transduce cells by crossing cell membranes, trafficking through endosomes to the cell nucleus, and delivering the DNA cargo of choice. Furthermore, by taking advantage of the tissue-specific tropism of different AAV serotypes, tissue-specific promoters, and route of administration, AAV vectors can be directed to transduce and express only in the desired retinal cell-type(s).

AAV gene augmentation vectors are therefore, in effect, complex biologic drugs that consist of a formulation (viral capsid), which encapsulates a pre-pro-drug (DNA), which is transcribed inside host cells into a pro-drug (RNA), which is translated and processed into the final biologic drug (protein). The product developmental lifecycles for these complex biologics present multiple challenges in terms of optimizing each of these stages of the drug. Moreover, product development also presents the challenge of considering host biological responses. To the host cells and immune system, the vector formulation presents foreign DNA sequences, viral expression elements, and viral capsid proteins, which cells will recognize both as nonspecific danger signals (through pathogen-associated molecular patterns and damage-associated molecular patterns) and as foreign antigens. As a result, both nonspecific clinical inflammation and specific acquired immunity may neutralize the vector effect, reduce cell viability, and further fuel immune responses. The current clinical toolbox for handling inflammation and immune responses typically revolves around companion steroid therapy.

The workshop's goals were therefore to develop a deeper understanding of physiological ocular immunity and immune activation in response to gene therapy; to discuss the prevalence and significance of inflammation during gene therapy for eye disease; to consider whether ocular inflammation and immune responses limit gene therapy's potential; and to identify knowledge gaps for future research.

## Premeeting Activities and Survey Results

As noted above, the premeeting survey sent to all participants posed 13 questions about preclinical studies and 13 questions about clinical studies. Survey questions sought information about experience with intraocular inflammation, including monitoring, treatment, and relationship to outcomes; screening for anti-AAV capsid antibodies in either animals or people before gene therapy treatment; development of antibody and cell-mediated responses to either the vector or the gene product; and use of immunosuppression. Sixteen of 18 survey recipients responded, and a summary of the results indicated the following:

• Inflammation during ocular gene therapy is not rare, but the severity of inflammation varied widely among both animal and clinical studies.
• In more severe or prolonged cases, inflammation was associated with reduced gene therapy efficacy.
• Inflammatory treatment regimens varied within and between clinical trials, with no clear consensus on approaches, and the effectiveness of treatment for inflammation was variable or unknown.

To set a baseline understanding of viral and ocular immunology in both normal and degenerating eyes, three introductory video presentations were posted online for all participants. Dr. Kai Chan provided an overview of the immune system and the nature of immune responses to AAV.^6^^,^^7^ Immune cells, including dendritic cells and macrophages, are equipped to recognize foreign molecular patterns using pattern recognition receptors such as Toll-like receptors (TLRs). These cells can mediate innate immune responses to AAV within hours or days. Subsequently, anti-capsid adaptive immune responses by B and T lymphocytes develop within days to weeks after AAV vector administration. Because wild-type AAVs are common natural infections, up to 70% of people have anti-AAV antibodies against various serotypes in their blood before treatment. Measurements of antibody responses to AAV using sera do not necessarily reflect immune responses within the eye, and immune responses to either the AAV vector or to the transgene product itself within the eye are difficult to study. Moreover, detecting subclinical inflammation in the eye depends on the sensitivity of available methods, which is often limited.

Dr. Thomas Langmann focused on the role of microglia, which are resident macrophages in the retina. Microglia contribute to immune responses in both negative and positive ways: these migratory cells actively clean up tissue debris but have also been associated with eye pathologies.^8^ Moreover, modulation of microglia responses with pharmacological agents can limit the extent and progression of retinal degeneration in different animal models, but data in human patients are rare.^9^ Preliminary data also suggest that microglia rapidly respond to ocular AAV.

Dr. Andrew Dick reviewed the complicated topic of immune privilege within the eye. Conventional wisdom has considered the eye to be sequestered from systemic immunity. In fact, the eye has extensive immune regulatory networks to maintain tissue homeostasis. These networks encompass a variety of both traditional immune cells (e.g., microglia/macrophages and lymphocytes) and noncanonical cells with immune activities that also contribute to intraocular immune regulation (e.g., retinal pigment epithelium [RPE]). Age, disease, and infection—including additional activation stimuli from AAV vector infection during gene therapy—all perturb these networks, potentially with important clinical consequences.^10^^–^^13^

## Lessons From Preclinical Animal Studies of Ocular Gene Therapy

### Meeting Presentations on Preclinical Studies

Ocular gene therapies have been tested by using a variety of animal models, including mice, rabbits, dogs, sheep, pigs, and several types of non-human primates (NHPs). Short presentations summarized both accumulated experience and current inflammation-related research from animal studies. Properties of the specific vectors discussed in all presentations are outlined in Tables 1A and 1B, and immunosuppression treatments used are outlined in Tables 2A and 2B.

**Table 1A.** Range of Properties for Ocular Gene Therapy Vectors Used in Studies Presented in the Workshop^*^: Preclinical Studies

Animal, PI	Vector: Serotype, Promoter, Transgene	Other Vector Elements	Codon Optimized?	CpG Depleted?	Dose, Volume, Route	Target Cells	Inflammation Noted	Comments	Refs
C57BL/6 mice, Pepple	AAV2 or AAV2-7m8.PR2.1.GFP	WPRE, β-globin/IgG chimeric intron	No	No	10^9^ – 10^11^ vg/eye, 1.0 – 1.5 µL, intravitreal	Rods	Clinical—visible in vivo	Clinical inflammation determined using OCT	19; ^†^
C57BL/6, CD-1 mice, Cepko	AAV8, AAV5, Anc80, AAV2-7m8; CMV, CAG, hUbiC, hBest1; GFP or no transgene	Variable	No	No	5 × 10^8^ – 10^9^ vg, subretinal	Cones, RPE	Subclinical	Dose-related effects on cones and RPE	14
NHP, *M. fascicularis*, Boye	AAV5.hGRK1.GUCY2D, AAV5.hGRK1.GFP	bGH	No	No	1.5 × 10^10^ – 1.5 × 10^11^ vg, 150 µL, subretinal	Cones, rods	Clinical—visible in vivo	Findings considered iatrogenic, non-adverse, had no impact on animal health or retinal function	^†^
RPGR dog (XLPRA2), Beltran	AAV2tYF.hGRK1.RPGRc		Yes	No	45 × 10^10^ vg, 150 µL, subretinal	PRs	Clinical—visible in vivo		20
RPGR dog (XLPRA2), Beltran	AAV2tYF.hGRK1.RPGR		Yes	No	9 × 10^10^ – 45 × 10^10^ vg, 150 µL, subretinal	PRs	Subclinical		20
RPGR dog (XLPRA2), Beltran	AAV2tYF.hGRK1.RPGRstb		No	No	45 × 10^10^ vg, 150 µL, subretinal	PRs	Clinical—visible in vivo		20
RHO^T4R/+^ dog, Beltran	AAV5.mOP.Rz525		No	No	150 × 10^10^ vg, 150 µL, subretinal	PRs	Clinical—visible in vivo; and/or subclinical		21
NHP, *M. fascicularis*, Merigan/McGregor	AAV2.CAG.GCaMP, AAV2.CAG. ChrimsonR.tdTomato				10^12^ – 3 × 10^14^ vg intravitreal	Retinal Ganglion	Clinical—visible in vivo		15
NHP, *M. fascicularis*, Fischer	AAV8.ARR3.CNGA3	WRPE^+^, bGH	No	No	10^11^ – 10^12^ vg, 200 µL, subretinal	Cones	Subclinical		22–24
NHP, *M. fascicularis*, Fischer	AAV8.phRHO.PDE6A	WRPE^+^, bGH	No	No	10^11^ – 10^12^ vg, subretinal	Rods	Subclinical		
Pigs, outbred wild-type, Chan	AAV8.CMV. eGFP	β-globin intron; β-globin poly(A)	Yes	No	4 × 10^10^ vg, 75 µL, subretinal	PRs, RPE	Clinical—visible in vivo; and/or subclinical	Several eyes without clinical inflammation had subclinical inflammation and/or pathology	16

bGH, bovine growth hormone polyadenylation signal; NHP, nonhuman primate; OCT, optical coherence tomography; PR, photoreceptors; Refs, references (if available); RPE, retinal pigment epithelium; RPGRc, codon optimized retinitis pigmentosa GTPase regulator (X-linked retinitis pigmentosa); RPGRstb, RPGR stabilized; WPRE, Woodchuck hepatitis virus (WHV) posttranslational regulatory element; XLRS, X-linked retinoschisis; **^+^**, WPRE modified with a point mutation in the ATG codon of the WHV-X open reading frame.

^*^The major characteristics of the AAV vectors used in studies presented during the workshop are shown.

^†^Unpublished data, 2020, discussed at the workshop.

**Table 1B.** Range of Properties for Ocular Gene Therapy Vectors Used in Studies Presented in the Workshop^*^: Clinical Studies

Disease, PI	Vector: Serotype, Promoter, Transgene	Other Elements	Codon Optimized?	CpG Depleted?	Dose, Volume, Route	Target Cells	Inflammation Noted	Comments	Refs
Choroideremia, Xue/MacLaren	AAV2.CAG.hREP1	WPRE, bGH	No	No	10^10^ – 10^11^ gp/mL, 100 µL subretinal	RPE	Clinical—visible in vivo	High dose	27
RPGR, Xue/MacLaren	AAV8.RK.coRPGR	bGH	Yes	No	5 × 10^10^ – 5 × 10^12^ gp/mL, up to 100 µL subretinal	PRs	Clinical—visible in vivo	High dose	28
XLRS, Sieving	AAV8.IRBP.hRS1.RS1		Yes	No	10^9^ – 10^11^ vg/eye, 50 µL intravitreal	Rods	Clinical—visible in vivo	Inflammation variable among high dose	29
XLRP, Shearman	AAV2tYF.GRK1.RPGR	SV40	Yes	Yes	Subretinal	PRs	Clinical—visible in vivo	Generally mild to moderate	^†^
Choroideremia, MacDonald	AAV2.CMV.CBA.CHM	WPRE, bGH	Yes	No	10^11^ vg, 100 µL subretinal	RPE	Clinical—visible in vivo		30
Achromatopsia, Fischer	AAV8.ARR3.CNGA3	WRPE, bGH	No	No	10^11^ – 5 × 10^11^vg, 200 µL subretinal	Cones	Clinical—visible in vivo	One case of transient iritis	26

bGH, bovine growth hormone polyadenylation signal; NHP, nonhuman primate; PR, photoreceptors; Refs, references (if available); RPE, retinal pigment epithelium; coRPGR, codon optimized retinitis pigmentosa GTPAse regulator (X-linked retinitis pigmentosa); WPRE, Woodchuck hepatitis virus (WHV) posttranslational regulatory element; XLRS, X-linked retinoschisis; **^+^**, WPRE modified with a point mutation in the ATG codon of the WHV-X open reading frame.

^*^The major characteristics of the AAV vectors used in studies presented during the workshop are shown.

^†^Unpublished data, 2020, discussed at the workshop.

**Table 2A.** Range of Ocular Gene Therapy Immunosuppression Protocols Used in Studies Presented In the Workshop:^*^ Preclinical Studies

Animals, PI	Tx Before Surgery: Dose, Agent, Route, Time	Tx During Surgery: Dose, Agent, Route	Tx After Surgery: Dose, Agent, Route, Time	Outcome—Inflammation	Outcome—Visual Function	Comments	Refs
NHP, *M. fascicularis*, Boye	Yes: prednisolone, oral, beginning 3 days before	Yes: 2 mg dexamethasone, subconjunctival	Yes: prednisolone, oral, continued for 6 weeks after	Test article related findings considered non-adverse; generally minimal to slight. At all doses, inflammatory findings in some animals (gray-white foci on ophthalmic exam) considered immune-mediated, based on their corresponding positive anti-AAV5 antibody results	ERG unaffected	No apparent difference noted between groups administered systemic steroids for an extended period, compared to those given short course	^†^
NHP, *M. fascicularis*, Boye	Yes: prednisolone, oral, beginning 1 day prior	Yes; 2 mg, dexamethasone, subconjunctival	Yes: prednisolone, oral, continued through 3 days after	Test article related findings considered nonadverse; generally minimal to slight. At all doses, inflammatory findings in some animals (gray-white foci on ophthalmic exam) considered immune-mediated, based on their corresponding positive anti-AAV5 antibody results	ERG unaffected	No apparent difference noted between groups administered systemic steroids for an extended period, compared to those given short course	^†^
Dog, Beltran	Yes: 1 mg/kg prednisone, oral, once before	Yes: 0.1% dexamethasone, topical	Yes: 1 mg/kg prednisone BID for 2 weeks, then 1 mg/kg SID for 2 weeks	Subclinical at lower doses, but increased to clinically apparent with higher dose	Visual behavior, ERG	Included ophthalmic examination, fundus photograph, cSLO/OCT	^†^
Dog, Beltran	Yes: 0.03% flurbiprofen, topical, 1 drop 3 times before	Yes: 2-4 mg triamcinolone acetonide, subconjunctival	Yes: 2-4 mg triamcinolone acetonide, subconjunctival once 4 weeks after	Subclinical at lower doses, but increased to clinically apparent with higher dose	Visual behavior, ERG	Included ophthalmic examination, fundus photograph, cSLO/OCT	31
NHP, *M. fascicularis*, Merigan/McGregor	Yes: 1 week to 5 months, sub-cutaneous cyclos-porine A; blood trough level 150-200 ng/ml	Yes: continued cyclosporine A	Yes: continued cyclosporine A	Inflammation occurred several weeks after injection, presumably related to high levels of transgene expression		Without immune suppression or when suppression stopped, high levels of transgene expression led to catastrophic loss of RGCs	^†^
Mice, Rpe65^−/−^, Lca5^gt/gt^, Rd1, Rd10, Rho^−/−^, Bennett	No	No	Yes, Pred-G ointment, topical, once	None	Safety, efficacy studies		32–35
RPE65^−/−^ dogs, Bennett	No	No	Yes, 40 mg Kenalog (triamcinolone acetonide) subconjunctival, once; Pred-G ointment, topical, once	None	Efficacy study		32, 36–39
Rcd1 dogs, Bennett/Cepko	No	No	Yes, 40 mg Kenalog (triamcinolone acetonide) subconjunctival, once; PredG ointment, topical, once	Severe inflammation		AAV.Anc80	^†^
NHP, *M. fascicularis*, *M. mulatta*, Bennett	No	No	Yes: 40 mg Kenalog (triamcinolone acetonide) subconjunctival, once; PredG ointment, topical, once	None	Safety study		40
NHP, *M. fascicularis*, CNGA3, Fischer	Yes: 1 mg/kg prednisolone, intramuscular, 2 days	Yes: 125 mg cefuroxime and 4 mg dexamethasone, subconjunctival	Yes: Dexamytrex and dexa-gentamicin, 3x times daily, 1 week; 1 mg/kg prednisolone, intramuscular, 5 days	No clinical inflammation related to test item; subclinical mononuclear inflammatory cell infiltrates in retina and choroid	No functional outcomes tested	NOAEL was above 1 × 10^12^ vg	^†^
NHP, *M. fascicularis*, PDE6A, Fischer	Yes: 1 mg/kg prednisolone, intramuscular, 2 days	Yes: 125 mg cefuroxime and 4 mg dexamethasone, subconjunctival	Yes: Dexamytrex and dexa-gentamicin, 3× times daily, 1 week; 1 mg/kg prednisolone, intramuscular, 5 days	No clinical inflammation related to test item; subclinical mononuclear inflammatory cell infiltrates in retina and choroid	No functional outcomes tested	NOAEL for ocular toxicity was 1 × 10^11^ vg; NOAEL for systemic toxicity was above 1 × 10^12^ vg	^†^
Pigs, outbred wild-type, Chan	No	Antibiotic and steroid ointment topically administered at end of surgery	No	Clinical inflammation (severe vitritis) in 1 of 5 eyes receiving unmodified vector beginning 2 weeks after. Subclinical inflammation in 2 of 5 eyes and PR pathology by histology in 5 of 5 eyes receiving unmodified vector at 6 week terminus (see text). No clinical signs in any of 5 eyes receiving TLR9-inhibitory modified vector; subclinical inflammation or pathology absent or reduced	OCT imaging supported histological findings. No ERG was performed.		16

cSLO/OCT, combined confocal scanning laser ophthalmoscope and optical coherence tomography; ERG, electroretinography; NHP, nonhuman primate; NOAEL, no adverse effect level; OCT, optical coherence tomography; Refs, references (if available); RPGR, X-linked retinitis pigmentosa; XLRS, X-linked retinoschisis.

^*^The major characteristics of the immunosuppression approaches used in studies presented during the workshop are shown.

^†^Unpublished data, 2020, discussed at the workshop.

**Table 2B.** Range of Ocular Gene Therapy Immunosuppression Protocols Used in Studies Presented in the Workshop:^*^ Clinical Studies

Disease, PI	Tx Before Surgery; Dose, Agent, Route, Time	Tx During Surgery; Dose, Agent, Route	Tx After Surgery; Dose, Agent, Route, Time	Outcome— Inflammation	Outcome—Visual Function	Comments	Refs
Choroideremia, Xue/MacLaren	Yes: 1 mg/kg, prednisolone, oral, starting 3 days before	No	Yes: First 7 low-dose patients: 1 mg/kg for 7 days after, then stopped. Protocol amended after a case of inflammation, for 7 high-dose patients: 1 mg/kg (day 3–7), 0.5 mg/kg (days 8–14), 0.25 mg/kg (days 15–16), 0.125 mg/kg (days 17–18), then stopped	1 case (out of 14) of intraocular inflammation seen at 2 weeks (after cessation of prednisolone) with vitreous cells, outer retinal opacities, and choroidal thickening	Visual acuity significantly reduced, but subsequently partially recovered after a course of oral prednisolone		27
RPGR, Xue/MacLaren	Yes: 1 mg/kg, prednisolone, oral, starting 3 days before	No	Yes: 1 mg/kg (day 3–7), 0.5 mg/kg (days 8–14), 0.25 mg/kg (days 15–16), 0.125 mg/kg (days 17–18), then stop.	7 cases (out of 18) of intraocular inflammation seen at higher end of dose escalation (1–5 × 10^12^ gp/mL), characterized by subretinal retinal infiltrates	Retinal sensitivity deteriorated with onset of inflammation, but recovered with corticosteroid therapy (systemic or local)		28
XLRS, Sieving	Yes: 60 mg oral prednisone, starting 2 days before	No	Yes: 60 mg oral prednisone, tapering over 2 months	2+ vitreous cells, 3+ anterior chamber, cell resolved by 6 months	Returned to baseline	Intravitreal dose 1 × 10^11^ vg/eye	29
Choroideremia, MacDonald	Yes: 1 mg/kg/d prednisone, oral 2 days	Yes: 1 mg/kg/d prednisone, oral	Yes: 1 mg/kg/d prednisone, oral 7 days, 0.5 next 7 days, 0.25 next 2 days, 0.125 next 2 days, then stopped	Vitreous inflammation (1 patient)	Loss of vision (1 patient)	Loss of central autofluorescence area on imaging (1 patient)	30
Achromatopsia, Fischer	Yes: 0.5% moxifloxacin drops and 0.5% dexamethasone gel, 4 times daily, 1 day; 1 mg/kg prednisolone, oral, 1 day	No	Yes: 0.5% moxifloxacin drops and 0.5% dexamethasone gel, 4 times daily, 21 days; 1 mg/kg prednisolone, oral, 20 days, tapered off after day 19	One case of very mild iritis, otherwise no inflammation	Improvement		^†^
Choroideremia (REP1), Fischer	Yes: 1 mg/kg prednisolone, oral, 2 days	No	Yes: 0.5% moxifloxacin drops and 0.5% dexamethasone gel, 4 times daily, 14–21 days; 1 mg/kg prednisolone, oral, 10 days, tapered off after day 10, total of 21 days	One case of pronounced postoperative vitritis; otherwise no test item-related inflammation			^†^
Retinitis pigmentosa (PDE6A), Fischer	Yes: 1 mg/kg prednisolone 1 mg/kg, 1 day	No	Yes: 0.5% moxifloxacin drops and 0.5% dexamethasone gel, 4 times daily, 14–21 days; 1 mg/kg prednisolone, oral, 10 days, tapered off after day 10, total of 21 days	No test item-related inflammation so far			^†^

NHP, nonhuman primate; Refs, references (if available); RPGR, X-linked retinitis pigmentosa; XLRS, X-linked retinoschisis.

^*^The major characteristics of the immunosuppression approaches used in studies presented during the workshop are shown.

^†^Unpublished data, 2020, discussed at the workshop.

Using inbred mice, Dr. Kathryn Pepple discussed the development of a mouse model of gene therapy–associated uveitis (GTAU) to study mechanisms and therapies. In naïve and AAV-exposed animals, intravitreal administration of an AAV2 vector (Table 1A) for treating red-green color blindness resulted in clinically evident inflammation in injected eyes within a week. Vitritis largely resolved by about one month. However, subclinical CD45^+^ cell infiltration was more persistent and was dominated by T cells. In AAV-exposed mice given intravitreal AAV injections, the timing of adaptive immune cell responses was accelerated, but the final cell composition was similar to that found in naïve animals.

Dr. Connie Cepko also studied inbred mice treated at birth with a variety of AAV vectors (Table 1A), using subretinal injections, to broadly assess vector toxicity with a large panel of cellular and functional assays.^14^ Results indicated that damage to cone outer segments and RPE after injection of AAV constructs, including empty vectors, tracked with dose. In contrast, damage did not correspond with other variables such as AAV capsid types, protein expression, protein contamination of preparations, or vector product status. Strikingly, however, toxicity correlated with using promoters that are active in RPE. Studies in dogs with retinitis pigmentosa gene defects revealed similar problems. Dog pups that were given subretinal injections with the artificial ancestral AAV-Anc80.GFP vector, which included a CMV promotor active in RPE, all had substantial eye inflammation within a month after injection (although the effects from the capsid cannot yet be distinguished from the effects of the promoter). Inflammation was difficult to control in some dogs, and severe inflammation corresponded with reduced efficacy as reflected by loss of GFP expression. Of note, in these mouse and dog studies, no steroid or other immunosuppressive treatments were used before AAV injection.

To develop more efficient AAV vectors for intravitreal injections, Dr. John Flannery described studies to generate AAV viral capsid variants by directed evolution. This work used naïve mice and cynomolgus macaques, which were not treated with steroids or other immunosuppression, to conduct short duration iterative screening of a library of AAV variants. The library was first screened for improved AAV penetration into the retina, and then the best variants were further screened for those that did not react with patient anti-AAV antisera. Finally, promising variants were tested individually in NHPs, retaining those that induced the lowest inflammatory responses. Interestingly, no inflammation in the eye was noted during the screening steps; this result may be due to using viruses that lacked an exogenous promoter and transgene construct or to the heterogeneity of the library such that no one variant was represented in sufficient quantity to induce immune responses.

Dr. Shannon Boye summarized findings from studying different routes of AAV administration. Toxicology and dose-ranging studies performed in support of clinical trials for Leber congenital amaurosis resulting from biallelic mutations in *GUCY2D* (LCA1) included mice with these mutations and wild-type cynomolgus macaques; animals received subretinal injections of AAV5-GRK1-GUCY2D or AAV5-GRK1-GFP, respectively (Table 1A). Steroids were administered one to three days before vector injection and continued for either three days or six weeks, with similar outcomes (Table 2A). Results from a separate NHP study demonstrated that combining subretinal and intravitreal delivery of AAV vectors carrying reporter genes (GFP/mCherry) in a single injection led to inflammation and loss of reporter expression over time. Novel AAV capsids for intravitreal delivery are now being designed to evade neutralizing antibodies. Finally, a study in beagles evaluating intracameral injection indicated that effective doses were associated with substantial inflammation and uveitis. Taken together, subretinal injection was the best tolerated of all injection routes.

Dr. William Beltran also described experience with subretinal or intravitreal injection with AAV2/5 vectors in dogs (Table 1A); these studies used immunosuppressive protocols initiated at the time of vector administration and that were continued for several weeks (Table 2A). Notably, lower AAV2/5 doses resulted only in subclinical inflammation, as reflected by subtle histological changes. Clinically apparent inflammation and anti-AAV capsid neutralizing antibodies (NAbs) increased with viral dose. Nonetheless, when the contralateral eye was treated several weeks after the first subretinal injection, the presence of high titers of NAbs directed against the AAV capsid did not prevent transgene expression. Overall, dose-ranging studies supported the idea of a therapeutic window, in which a dose could be identified that was high enough to provide effective treatment but below a range that induced excessive inflammation at cellular and tissue levels.

Dr. Scott Ellis described toxicology studies in mice and cynomolgus macaques after a single subretinal injection of GT005, an AAV vector designed to treat dry age-related macular degeneration by expressing human complement factor I. In the macaque study, animals were given intramuscular steroids one day before treatment and subsequently treated with systemic steroids if symptoms developed. In these studies, inflammatory responses in treated eyes were frequent, dose-dependent and, in the primate study, corresponded with the development of an anti-transgene response to the human complement factor I protein. In contrast, inflammation did not track with antivector antibodies. The responses therefore appeared species-specific. Overall, preclinical studies of GT005 raised no safety concerns, and GT005 is now being evaluated in Phase II clinical trials in patients with geographic atrophy (GA) caused by dry AMD.

Dr. Juliette McGregor and Dr. William Merigan presented data from imaging studies in cynomolgus NHPs (*Macaca fascicularis*) that used adaptive optics to optically record from retinal ganglion cells following intravitreal injection of AAV2 vectors (Table 1A) for calcium imaging (GCaMP) optogenetic therapy (ChrimsonR).^15^ Because inflammation would greatly limit imaging, macaques were both pre-screened to exclude animals with anti-AAV antibodies and pretreated with subcutaneous cyclosporine A for one to 20 weeks before AAV vector injection (Table 2A). Although numbers were small, increasing time of immunosuppressive pretreatment was generally associated with improved lateral extent of foveal gene expression. In the absence of immunosuppression, transgene expression was unpredictable and strong expression was often transient. If inflammation developed due to transgene expression, corticosteroids were administered by intravitreal injection.

Mice, rabbits, dogs, cynomolgus macaques (*Macaca fascicularis*), and rhesus macaques (*Macaca mulatta*) were used during development of Luxturna, now licensed in the United States and indicated for treatment of people with mutations in the RPE65 gene. Summarizing experience across many studies (Tables 1A and 1B and Tables 2A and 2B), Dr. Jean Bennett concluded that overt clinical inflammation was rare, but histopathological findings, such as focal changes in the RPE (hyperplasia and hypertrophy), scattered mononuclear cells in the vitreous, focal choroiditis under the subretinal injection site, and scar formation at the retinotomy site in retinas were common, although generally mild. Several variables were associated with the development of inflammation, including dose, AAV serotype, route, vector production, and time. The development of NAbs to AAV capsids was variable but not obviously related to outcomes, and the fellow eye of NHPs who developed NAbs after subretinal injections could be retreated successfully.

Dr. Dominik Fischer described data from studies using cynomolgus NHPs (*Macaca fascicularis*) that compared subretinal or intravitreal injection of an AAV8 vector expressing CNGA3 (Tables 1A and 1B). Immune modulation included systemic steroids given for seven days, starting two days before surgery, and local steroids at surgery and for one week thereafter (Tables 2A and 2B). Subretinal injection was not associated with development of anti-AAV8 antibodies, but intravitreal administration induced substantial serum antibody levels. By 90 days after treatment, retinas from NHPs treated with 10^12^ vector genomes (vg) did not exhibit adverse reactions. Interestingly, a follow-up study with sacrifice at 28 days did reveal perivascular lymphocytic infiltration at the highest dose level (10^12^ vg), which was then deemed transient. Similar to findings in NHPs, achromatopsia patients treated by subretinal injection with AAV2/8CNGA3 did not develop anti-AAV8 antibodies. To aid in evaluating vector toxicity, several in vitro methods to test toxicity are being developed. These include the use of PMA-differentiated human monocyte THP-1 cell cultures that express TLRs, and thus the cells produce cytokines on TLR or inflammasome activation by vector preparations.

AAV is a DNA virus, and thus TLR9, which senses endosomal DNA, has particularly been implicated in AAV gene therapy–related inflammation. To develop a vector-intrinsic strategy against TLR9-mediated inflammation, Dr. Kai Chan constructed two AAV8 vectors expressing GFP (Table 1A). One was an unmodified vector, and one added a nontranslated telomeric DNA sequence outside of the protein coding region, dubbed io2, designed to blunt TLR9 activation. Because the goal was to characterize and compare immune responses, the vectors were compared in a pig model^16^ by using subretinal injections at a higher AAV dose and without immunosuppression (Table 2A). Among eyes injected with the unmodified AAV8.GFP vector, one of five exhibited clinical inflammation in the form of vitritis during the in-life phase. However, after euthanasia at six weeks after injection, histological studies on the retinas revealed pathological changes in all five eyes. Changes included shortening or loss of cone photoreceptor outer segments, as well as increased infiltration by microglia in the outer nuclear layer and CD8^+^ T cell infiltration in the retina. In contrast, these detrimental changes were substantially reduced or not detected in the fellow eyes of the same five animals given the AAV8.GFP.io2 vector, and none of those five eyes exhibited vitritis during the in-life phase.^16^

### Group Discussion of Preclinical Presentations

The experience with the extent of subclinical inflammation in animal studies was a substantial point of discussion. Comments noted the following:

• Even with subretinal injection, ocular inflammation was found in almost all animal studies, and the degree of inflammation appeared to be related to dose.
• Animal studies often uncovered histological changes and cellular infiltrations in the absence of clinically apparent inflammation. However, approaches that detect clinical inflammation in animal eyes are insensitive and thus may miss persistent underlying inflammation.
• NHPs were considered the best available option for identifying “no observed adverse-effect levels” and for guiding dose selection in people, but issues associated with NHP recognition of human transgenes also limited translational relevance.

Discussions of the impact of inflammation on efficacy included the following comments:

• In several examples, pre-existing or induced anti-AAV antibodies apparently did not impact efficacy.
• On the other hand, in some cases antitransgene antibodies were associated with reduced effectiveness.
• Although potentially feasible in animal models, many preclinical studies have not evaluated the degree of transgene expression or assessed immune responses to the transgene, limiting conclusions about impact on efficacy.

The relevance of animal models in general was discussed, with the following observations:

• By necessity, the design of animal experiments often has important differences from clinical situations. Laboratory animals, even when outbred, have less diverse genetic backgrounds that may not accurately reflect human genetic diversity. Different animals also inherently exhibit differential sensitivities to AAV injections (e.g., dogs seem to be the most sensitive and mice the least).
• Many preclinical studies are performed using healthy animals, but people being treated for IRDs have underlying disease typically accompanied by cell loss and baseline inflammation.
• The use of immunosuppressive pretreatment varied widely in different animal studies, from none to substantial, but pretreatment strategies are not well aligned with clinical protocols. Equally important, the question of how well immunosuppressive treatments actually work has not been systematically studied, and the critical immunosuppressive mechanisms involved have not been determined.

Overall, species differences coupled with the variability in study designs made it difficult to interpret how well different animal models reflect ocular gene therapy outcomes in people.

## Lessons From Clinical Studies of Ocular Gene Therapy

### Meeting Presentations on Clinical Studies

The second day of the workshop focused on human clinical experience from gene therapy trials. Presentations were structured to consider whether inflammation, or managing inflammation, interferes with treatment goals, namely to deliver the optimal formulation and amount of gene therapy vector that yields the best protein expression and efficacy.

Dr. Christine Kay summarized clinical observations gained from work using a variety of protocols. She noted that eyes with IRDs exist in a state of low-grade inflammation, and surgery itself is inflammatory. AAV treatment–related inflammation was typical after either subretinal or intravitreal injections and appears to be dose dependent. Across different clinical protocols, surgical techniques vary, dose and volume of AAV delivered varies, and the type and duration of presurgery immunosuppression varies. Technical differences such as the use of pre-blebs, bleb location, and volume of vector delivered may have substantial implications for interpreting dose-escalation studies. Anecdotally, Dr. Kay estimated that about 10% of gene therapy patients have significant clinically apparent inflammation that can be treated with either oral or periocular steroids, but a considerably larger percentage of patients appear to have subtle, subclinical inflammation. In some cases, this inflammation can be detected by fluorescein angiography and optical coherence tomography; loss of retinal function can be detected with microperimetry and occasionally by visual acuity measurements. Despite treatment, some inflammation can be long-lasting and appears to correlate with loss of efficacy.

On the basis of his experience, as well as literature reports, Dr. Tim Stout estimated that as much as 25% to 35% of patients have some degree of GTAU, more frequently following intravitreal injection than after subretinal injection. Dr. Stout's protocols typically use oral steroid pretreatment ranging from three to seven days before surgery, coupled with local steroid treatment during and after surgery. Whereas most inflammation seems to be transient and treatable, its impact on transgene expression has not been clear. A number of factors may promote inflammation, including viral dose and transduction kinetics. To better understand the role of dose, Drs. Stout and Violet Lin modeled the subretinal injection site mathematically to estimate the multiplicity of infection (MOI) of viral particles per cell in the retina. In their experience, surgical blebs are typically either a semisphere shape or a squashed-sphere shape; 300 µL macular blebs average about 14 mm in diameter, making contact with as many as 18 × 10^6^ cells. Using bleb morphology and diameter coupled with assumptions about volume, cell density, and retinal dimensions, preliminary modeling results suggested that AAV MOIs could be quite large. Estimated MOIs ranged from about 9600:1 for rods to 212,000:1 for RPE cells. These calculations raised concerns about vector overtreatment and potential off-target effects.

Dose-escalation studies of intravitreal injection with GS010 (Lumevoq) for treating Leber hereditary optic neuropathy, described by Dr. José-Alain Sahel, did not use immunosuppression before surgery. Inflammation was seen in more than 90% of patients but was mostly mild and treatable; however, the use of high doses was limited by inflammation. Optogenetic trials of another product, GS030, to treat one form of retinitis pigmentosa did use immunosuppression at time of surgery; to date, mild but manageable intraocular inflammation has been observed. Patients have been monitored for serum anti-AAV antibodies at the time of treatment and for development of antibodies and T-cell responses, but so far immune status and treatment outcomes have not been related.

Dr. Kanmin Xue discussed clinical studies using subretinal injections of an AAV2 vector expressing REP1 to treat choroideremia and of an AAV8 vector expressing codon-optimized retinitis pigmentosa GTPase regulator gene (RPGR) to treat X-linked retinitis pigmentosa (Table 1B). Some patients developed signs of retinal inflammation that were related to vector dose and resolved with treatment (Table 2B). Because inflammation developed in the first patient to receive high-dose gene therapy for choroideremia, the standard perioperative immunosuppression regime was increased from seven days to 21 days. Close observation within the first three months after treatment was important, because signs of retinal inflammation may be subtle and correlate with fluctuations in macular function; timely intervention was important to improve clinical outcome. Laboratory studies suggested a potential role for hydroxychloroquine as an adjunct to suppress TLR9-mediated immune activation in the retina during innate immune responses after AAV gene therapy.

Dose-escalation studies of AAV8-RS1 to treat X-linked retinoschisis (Table 1B) indicated that inflammation increased with dose, as presented by Dr. Paul Sieving. Appropriate delivery of this transgene, a secreted protein, via intravitreal vector injection is a complex and challenging process. Initial surgeries used oral prednisone pretreatment (Table 2B). Although NAb responses among patients were variable, the sera of some patients contained substantial amounts of NAbs despite immunosuppression, and NAb levels appeared to track with inflammation. The most recent surgeries have therefore used a triple drug immunosuppression regimen (a combination of prednisone, cyclosporin, and mycophenolate mofetil).

Dr. Mark Shearman reviewed NHP and clinical studies (Table 1B) of AAV vectors designed to treat X-linked retinoschisis, X-linked retinitis pigmentosa, and two forms of achromatopsia. NHP studies intentionally did not use steroid pretreatment, but some animals were treated with steroids at surgery or when needed. Across different NHP studies, inflammation increased with dose but was transient, as reflected mostly by in-life clinical evaluations. Outcomes appeared to be improved by purifying AAV vector preparations to remove empty capsids. The presence of pre-existing anti-AAV serum antibodies, or development of anti-AAV antibodies on injection, did not correspond with the extent of ocular inflammation and did not prohibit gene expression. Only anti-AAV capsid antibodies have been observed, not antibodies to the transgenes. Clinical studies have used steroid treatment at the time of surgery and for a period after vector administration, tapering over time; to date, mild to moderate inflammation has been observed, and a few patients had development of GTAU.

Dr. Ian MacDonald described his experience to date with an AAV2 vector expressing REP1 to treat choroideremia (Table 1B), which used a 21-day steroid regimen, including 2 days’ pretreatment (Table 2B). Despite pretreatment, five of six patients had some level of intraocular inflammation. One subject had significant loss of the central macular retinal pigment epithelium, as revealed by fundus autofluorescence. The same patient experienced a serious adverse event after the initial steroid treatment stopped. Hyperreflective deposits appeared within the retina, presumably cellular infiltrates; these bodies resolved slowly with a second course of systemic steroid treatment. Dr. MacDonald noted that these patients were being treated at a relatively late disease stage, which may in itself limit both safety and efficacy outcomes.

### Group Discussion of Clinical Presentations

Similar to animal studies, large differences in clinical practice were noted: prophylactic and symptomatic immunosuppressive protocols, subretinal injection techniques, and treatment regimens after surgery all varied considerably. The relationship between viral dose, efficacy, and inflammation was a particular point of discussion, with commenters noting the following:

• The use of large MOIs was considered important, although viral loss during administration, empty capsids, and other factors likely reduce the calculated MOI to a lower effective MOI.
• Interpretations of calculated MOIs are complicated by the lack of a standardized assay to measure infectious viral particles and lack of understanding of the number of intracellular infectious particles required for optimal efficacy, not just in vitro assays of genome equivalents that are currently used to express viral titers. New research assays such as signal amplification by exchange reaction fluorescence in situ hybridization (SABER-FISH), which detects AAV genomes in tissues,^17^^,^^18^ might be leveraged to better quantitate infectious virus. Product potency assays may also be useful.
• Because transgene expression itself can only be monitored in people if the resulting protein can be detected in the anterior chamber, evaluating whether doses currently being used result in “overtreatment” is difficult. Applying adaptive optics imaging and incorporation of fluorescent reporters to understand gene expression and immune responses in situ in people may be helpful.

Inflammation has also been observed in uninjected fellow eyes after intravitreal administration of AAV, and in a few cases apparent efficacy has been observed. The mechanisms underlying such effects are not clear, although some NHP studies found the presence of vector in fellow eyes by PCR. These observations strongly suggest that using the status of the fellow eye as a control or comparator could be problematic.

## Summary of Discussion Outcomes: Workshop Themes

### Areas of Consensus

The workshop's discussions consistently suggested that, in both animals and people, ocular inflammation almost always accompanies gene therapy treatments by any route, and the degree of inflammation is correlated with dose. Beyond dose per se, other variables such as viral concentration, volume, and retinal surface area appear to be important. However, harmonizing these parameters between small-globe animals and people to study them is difficult. Even when inflammation was not clinically apparent, tissue and cellular changes in the retina were often found. Interpreting the significance of these changes is not straightforward.

While low levels of inflammation can be treated, severe inflammation both limits AAV dose and is consistently associated with reduced efficacy. Clearly a wide variety of different treatment protocols are being used before, during, and after gene therapy administration to suppress unwanted clinical inflammation, NAbs, or T cell responses. The heterogeneity in research and clinical protocols confounds conclusions about optimal strategies at this time. Identifying consensus protocols and best practices might help minimize study variables and allow harmonization, adding rigor to clinical studies. However, the amounts of cellular immune activation that could potentially reduce gene therapy efficacy are not clear, and correspondingly no evidence supports an approach to select the desired amounts of immunosuppression.

Immunosuppression regimens typically use various corticosteroids, locally or systemically, which is problematic; long-term steroid use, in particular, has undesirable consequences. The choice of steroid treatments or immunomodulatory therapy with cyclosporin A or other agents should be tailored in context, such as the nature of the patient's disease, age, and comorbidities, as well as the nature of the vector and route of administration. Using targeted biologics instead could be attractive, but the knowledge base to rationally choose targets and products is insufficient. This is an important area for future research. Some indication for using targeted immunomodulators, or perhaps biologics that inhibit lysosomal pathways and thus lysosomal TLR activation (such as through mechanistic target of rapamycin kinase (mTOR) inhibition), was supported by preclinical data presented on use of hydroxychloroquine.

### Areas of Divergence and Uncertainty

Despite considerable study, the relationship between pre-existing or induced immune responses to viral vectors and subsequent outcomes is still unclear. Several examples from animal and clinical studies suggested that treatment can be successful despite antivector antibody responses. Other data indicated that clinical or subclinical inflammation was associated with antivector or antitransgene immunity, potentially limiting dose or reducing benefits. The relative significance of inflammation, as well as adaptive immune responses, during clinical treatments therefore remained a matter of debate.

Issues related to vector manufacturing were noted repeatedly, as was the fact that no consensus has been developed regarding the preferred level of vector purification, the acceptable levels of contaminants and residuals, the methods of vector quantitation, or the methods of determining infectious virus particles. AAV vectors currently in clinical use have the momentum of history and past investment, but research is providing next-generation vectors and manufacturing improvements. Potential refinements include engineering less-inflammatory viral variants, improving promoters, incorporating immunomodulatory sequences, and removing empty capsids. The promising options are a reminder that future product development should not default to a “plug-and-play” mentality but take full advantage of all advances to optimize each insert and vector.

## Workshop Implications and Next Steps

Not surprisingly, the workshop discussions raised as many questions as they provided answers. In general, the workshop's deliberations implied that broad interdisciplinary approaches should be encouraged and supported to address these difficult questions with maximum efficiency. Potential actions and resources that could be addressed in the near term include the following:

• Establishing and fostering a consultant directory of subject matter experts who will be available to provide ocular-specific immunological expertise at any stage of animal or clinical studies. The goal of this directory would be to foster new collaborations and promote integrated evaluation of inflammation and immune responses into all ocular gene therapy studies.
• Establishing a central data repository to consolidate animal and clinical data pertaining to inflammation observed during any form of ocular gene therapy. Academic centers and companies, including discontinued programs and researchers with unpublished (negative) data, could be recruited to participate. Investigators could be invited to voluntarily deposit data, using a structured format that anonymizes patient and proprietary information. The premeeting survey used during this workshop illustrates the types of data of interest. The goal of the repository would be to facilitate analyses that compare many variables with therapy outcomes, thereby informing future research and identifying best clinical practices.
• Involving regulators such as those at Center for Biologics Evaluation and Research, U.S. Food and Drug Administration (CBER/FDA), who review clinical and adverse event data across many ocular gene therapy clinical trials and thus have unique experience and perspective, in these discussions. Of note, in December 2020, the National Center for Advancing Translational Sciences at the National Institutes of Health and CBER/FDA cohosted a Virtual Workshop on Systemic Immunogenicity Considerations for Adeno-Associated Virus (AAV)–Mediated Gene Therapy. On the basis of the findings from both workshops and findings in animal models, new regulatory guidance regarding managing inflammation throughout ocular gene therapy product development could be considered.
• Organizing a future, public meeting to continue these discussions, especially in the clinical space.

In the longer term, the workshop discussions identified major areas for future research. The questions raised and research responses include the following:

– What is the nature of inflammation and immune activation during retinal degeneration?
  • Research is needed to better understand each inherited retinal disease, particularly in terms of ongoing disease-related ocular inflammation present before gene therapy treatment. To enable evaluations, longitudinal blood and eye samples should be collected during clinical studies.
– What is the best use of inbred and outbred animal models?
  • Research is needed to better understand the strengths and weaknesses of small and large animal models of both IRDs and ocular gene therapy, in order to select those best suited to the purposes under study.
– What is the nature of inflammation and immune activation during ocular gene therapy? Research is needed to do the following:
  • Comprehensively quantitate inflammatory and adaptive immune responses to ocular gene therapy in vivo at the cell and tissue levels, within the eye as well as systemically. Inflammatory activation pathways and immunological functions of noncanonical cells, such as RPE, deserve particular attention.
  • Determine the localization and persistence of injected viral particles, both in terms of capsids and cargo.
  • Evaluate new in vitro assays that may inform clinical practice.

Of note, to enable all evaluations, longitudinal blood and eye samples should be collected during clinical studies.

– What is the best way to control inflammation before, during, and after AAV therapy, and what effects do anti-inflammatory treatments have on overall safety as well as the efficiency of in vivo gene expression? Research is needed to do the following:
  • Apply the latest insights from nonclinical animal models of inflammation after gene therapy to human studies and systematically apply nonclinical data to selecting optimal, safe, and effective immunosuppression regimens.
  • Support optimizing and harmonizing prophylactic and symptomatic treatment of inflammation in clinical protocols and develop treatments that are more nuanced than steroids.
  • Optimize vector gene expression and efficacy at the cell, tissue, and functional vision levels (beyond maximizing the multiplicity of infection).
  • Assess the impact of suppressing inflammation on transgene expression.
  • Develop new clinical imaging tools to better monitor subclinical cellular inflammatory responses in vivo.
– Can better viral vectors, particularly better viral promoters, be developed to deliver genes effectively without triggering immune responses? Research is needed to do the following:
  • Better understand the individual components of gene therapy products that stimulate inflammatory responses, such as the relative roles of capsid proteins, promoters, and genomic DNA (including 5′-cytosine-phosphoguanine dinucleotide (CpG) content).
  • Further evaluate whether certain promoters have unique roles in promoting inflammation, including research to independently evaluate recent findings indicating that promoters active in RPE are toxic.

Taken together, we hope these efforts have identified knowledge gaps and will serve to intensify focus on the underappreciated but critical role of ocular inflammation during gene therapies for IRDs. The insights resulting from the workshop have suggested research and clinical studies that can pinpoint best practices and accelerate cures for these blinding diseases. The Foundation intends to actively explore ways to support work in these critical areas.

## References

[1] Netea MG, Balkwill F, Chonchol M, et al. A guiding map for inflammation. *Nat Immunol*. 2017; 18: 826–831. 28722720 10.1038/ni.3790 PMC5939996

[2] Raper SE, Chirmule N, Lee FS, et al. Fatal systemic inflammatory response syndrome in a ornithine transcarbamylase deficient patient following adenoviral gene transfer. *Mol Genet Metab*. 2003; 80: 148–158. 14567964 10.1016/j.ymgme.2003.08.016

[3] Nienhuis AW, Dunbar CE, Sorrentino BP. Genotoxicity of retroviral integration in hematopoietic cells. *Mol Ther*. 2006; 13: 1031–1049. 16624621 10.1016/j.ymthe.2006.03.001

[4] Wang D, Tai PWL, Gao G. Adeno-associated virus vector as a platform for gene therapy delivery. *Nat Rev Drug Discov*. 2019; 18: 358–378. 30710128 10.1038/s41573-019-0012-9 PMC6927556

[5] Buck TM, Wijnholds J. Recombinant adeno-associated viral vectors (rAAV)-vector elements in ocular gene therapy clinical trials and transgene expression and bioactivity assays. *Int J Mol Sci*. 2020; 21:4197. 10.3390/ijms21124197 PMC7352801 32545533

[6] Rabinowitz J, Chan YK, Samulski RJ. Adeno-associated virus (AAV) versus immune response. *Viruses*. 2019; 11:102. 10.3390/v11020102 PMC6409805 30691064

[7] Verdera HC, Kuranda K, Mingozzi F. AAV vector immunogenicity in humans: a long journey to successful gene transfer. *Mol Ther*. 2020; 28: 723–746. 31972133 10.1016/j.ymthe.2019.12.010 PMC7054726

[8] Rashid K, Akhtar-Schaefer I, Langmann T. Microglia in retinal degeneration. *Front Immunol*. 2019; 10: 1975. 31481963 10.3389/fimmu.2019.01975 PMC6710350

[9] Akhtar-Schäfer I, Wang L, Krohne TU, Xu H, Langmann T. Modulation of three key innate immune pathways for the most common retinal degenerative diseases. *EMBO Mol Med*. 2018; 10:e8259. 10.15252/emmm.201708259 PMC6180304 30224384

[10] Copland DA, Theodoropoulou S, Liu J, Dick AD. A perspective of AMD through the eyes of immunology. *Invest Ophthalmol Vis Sci*. 2018; 59: Amd83–amd92. 30025105 10.1167/iovs.18-23893

[11] Dick AD. Doyne lecture 2016: intraocular health and the many faces of inflammation. *Eye (Lond)*. 2017; 31: 87–96. 27636226 10.1038/eye.2016.177 PMC5233925

[12] Forrester JV, Xu H. Good news-bad news: the Yin and Yang of immune privilege in the eye. *Front Immunol*. 2012; 3: 338. 23230433 10.3389/fimmu.2012.00338 PMC3515883

[13] Perez VL, Caspi RR. Immune mechanisms in inflammatory and degenerative eye disease. *Trends Immunol*. 2015; 36: 354–363. 25981967 10.1016/j.it.2015.04.003 PMC4563859

[14] Xiong W, Wu DM, Xue Y, et al. AAV cis-regulatory sequences are correlated with ocular toxicity. *Proc Natl Acad Sci USA*. 2019; 116: 5785–5794. 30833387 10.1073/pnas.1821000116 PMC6431174

[15] McGregor JE, Godat T, Dhakal KR, et al. Optogenetic restoration of retinal ganglion cell activity in the living primate. *Nat Commun*. 2020; 11: 1703. 32245977 10.1038/s41467-020-15317-6 PMC7125151

[16] Chan YK, Wang SK, Chu CJ, et al. Engineering adeno-associated viral vectors to evade innate immune and inflammatory responses. *Sci Transl Med*. 2021; 13(580): eabd3438. 33568518 10.1126/scitranslmed.abd3438 PMC8409505

[17] Wang SK, Lapan SW, Hong CM, Krause TB, Cepko CL. In situ detection of adeno-associated viral vector venomes with SABER-FISH. *Mol Ther Methods Clin Dev*. 2020; 19: 376–386. 33209963 10.1016/j.omtm.2020.10.003 PMC7658570

[18] Kishi JY, Lapan SW, Beliveau BJ, et al. SABER amplifies FISH: enhanced multiplexed imaging of RNA and DNA in cells and tissues. *Nat Methods*. 2019; 16: 533–544. 31110282 10.1038/s41592-019-0404-0 PMC6544483

[19] Mancuso K, Hauswirth WW, Li Q, et al. Gene therapy for red-green colour blindness in adult primates. *Nature*. 2009; 461: 784–787. 19759534 10.1038/nature08401 PMC2782927

[20] Song C, Dufour VL, Cideciyan AV, et al. Dose range finding studies with two RPGR transgenes in a canine model of X-linked retinitis pigmentosa treated with subretinal gene therapy. *Hum Gene Ther*. 2020; 31: 743–755. 32414297 10.1089/hum.2019.337 PMC7404825

[21] Cideciyan AV, Sudharsan R, Dufour VL, et al. Mutation-independent rhodopsin gene therapy by knockdown and replacement with a single AAV vector. *Proc Natl Acad Sci USA*. 2018; 115: E8547–e8556. 30127005 10.1073/pnas.1805055115 PMC6130384

[22] Reichel FF, Dauletbekov DL, Klein R, et al. AAV8 can induce innate and adaptive immune response in the primate eye. *Mol Ther*. 2017; 25: 2648–2660. 28970046 10.1016/j.ymthe.2017.08.018 PMC5768589

[23] Seitz IP, Michalakis S, Wilhelm B, et al. Superior retinal gene transfer and biodistribution profile of subretinal versus intravitreal delivery of AAV8 in nonhuman primates. *Invest Ophthalmol Vis Sci*. 2017; 58: 5792–5801. 29117317 10.1167/iovs.17-22473

[24] Reichel FF, Peters T, Wilhelm B, et al. Humoral immune response after intravitreal but not after subretinal AAV8 in primates and patients. *Invest Ophthalmol Vis Sci*. 2018; 59: 1910–1915. 29677353 10.1167/iovs.17-22494

[25] Fischer MD, Ochakovski GA, Beier B, et al. Efficacy and safety of retinal gene therapy using adeno-associated virus vector for patients with choroideremia: a randomized clinical trial. *JAMA Ophthalmol*. 2019; 137: 1247–1254. 10.1001/jamaophthalmol.2019.3278 PMC6865291 31465092

[26] Fischer MD, Michalakis S, Wilhelm B, et al. Safety and vision outcomes of subretinal gene therapy targeting cone photoreceptors in achromatopsia: a nonrandomized controlled trial. *JAMA Ophthalmol*. 2020; 138: 643–651. 32352493 10.1001/jamaophthalmol.2020.1032 PMC7193523

[27] Xue K, Jolly JK, Barnard AR, et al. Beneficial effects on vision in patients undergoing retinal gene therapy for choroideremia. *Nat Med*. 2018; 24: 1507–1512. 30297895 10.1038/s41591-018-0185-5 PMC7032956

[28] Cehajic-Kapetanovic J, Xue K, Martinez-Fernandez de la Camara C, et al. Initial results from a first-in-human gene therapy trial on X-linked retinitis pigmentosa caused by mutations in RPGR. *Nat Med*. 2020; 26: 354–359. 32094925 10.1038/s41591-020-0763-1 PMC7104347

[29] Cukras C, Wiley HE, Jeffrey BG, et al. Retinal AAV8-RS1 gene therapy for X-linked retinoschisis: initial findings from a phase I/IIa trial by intravitreal delivery. *Mol Ther*. 2018; 26: 2282–2294. 30196853 10.1016/j.ymthe.2018.05.025 PMC6127971

[30] Dimopoulos IS, Hoang SC, Radziwon A, et al. Two-year results after AAV2-mediated gene therapy for choroideremia: the Alberta experience. *Am J Ophthalmol*. 2018; 193: 130–142. 29940166 10.1016/j.ajo.2018.06.011

[31] Dufour VL, Cideciyan AV, Ye GJ, et al. Toxicity and efficacy evaluation of an adeno-associated virus vector expressing codon-optimized RPGR delivered by subretinal injection in a canine model of X-linked retinitis pigmentosa. *Hum Gene Ther*. 2020; 31: 253–267. 31910043 10.1089/hum.2019.297 PMC7047101

[32] Bennicelli J, Wright JF, Komaromy A, et al. Reversal of blindness in animal models of leber congenital amaurosis using optimized AAV2-mediated gene transfer. *Mol Ther*. 2008; 16: 458–465. 18209734 10.1038/sj.mt.6300389 PMC2842085

[33] Song JY, Aravand P, Nikonov S, et al. Amelioration of neurosensory structure and function in animal and cellular models of a congenital blindness. *Mol Ther*. 2018; 26: 1581–1593. 29673930 10.1016/j.ymthe.2018.03.015 PMC5986734

[34] McDougald DS, Dine KE, Zezulin AU, Bennett J, Shindler KS. SIRT1 and NRF2 gene transfer mediate distinct neuroprotective effects upon retinal ganglion cell survival and function in experimental optic neuritis. *Invest Ophthalmol Vis Sci*. 2018; 59: 1212–1220. 29494741 10.1167/iovs.17-22972 PMC5839257

[35] Uyhazi KE, Aravand P, Bell BA, et al. Treatment potential for LCA5-associated Leber congenital amaurosis. *Invest Ophthalmol Vis Sci*. 2020; 61: 30. 10.1167/iovs.61.5.30 PMC7405811 32428231

[36] Acland GM, Aguirre GD, Ray J, et al. Gene therapy restores vision in a canine model of childhood blindness. *Nat Genet*. 2001; 28: 92–95. 11326284 10.1038/ng0501-92

[37] Acland GM, Aguirre GD, Bennett J, et al. Long-term restoration of rod and cone vision by single dose rAAV-mediated gene transfer to the retina in a canine model of childhood blindness. *Mol Ther*. 2005; 12: 1072–1082. 16226919 10.1016/j.ymthe.2005.08.008 PMC3647373

[38] Jacobson SG, Acland GM, Aguirre GD, et al. Safety of recombinant adeno-associated virus type 2-RPE65 vector delivered by ocular subretinal injection. *Mol Ther*. 2006; 13: 1074–1084. 16644289 10.1016/j.ymthe.2006.03.005

[39] Amado D, Mingozzi F, Hui D, et al. Safety and efficacy of subretinal readministration of a viral vector in large animals to treat congenital blindness. *Sci Transl Med*. 2010; 2: 21ra16. 10.1126/scitranslmed.3000659 PMC4169124 20374996

[40] Weed L, Ammar MJ, Zhou S, et al. Safety of same-eye subretinal sequential readministration of AAV2-hRPE65v2 in non-human primates. *Mol Ther Methods Clin Dev*. 2019; 15: 133–148. 31660416 10.1016/j.omtm.2019.08.011 PMC6807311

